# Nitrogen Recovery from Landfill Leachate Using Lab- and Pilot-Scale Membrane Contactors: Research into Fouling Development and Membrane Characterization Effects

**DOI:** 10.3390/membranes12090837

**Published:** 2022-08-27

**Authors:** Ilaria Righetto, Raed A. Al-Juboori, Juho Uzkurt Kaljunen, Ngoc Huynh, Anna Mikola

**Affiliations:** 1 Department of Environment, Land and Infrastructure Engineering, Politecnico di Torino, Corso Duca degli Abruzzi, 24, 10129 Torino, Italy; 2 Water and Environmental Engineering Research Group, Department of Built Environment, Aalto University, P.O. Box 15200, Aalto, FI-00076 Espoo, Finland; 3 Department of Bioproducts and Biosystems, School of Chemical Engineering, Aalto University, P.O. Box 11000, Aalto, FI-00076 Espoo, Finland

**Keywords:** nutrient recovery, membrane contactor, landfill leachate, ammonia transfer rate, membrane fouling, tannins

## Abstract

Membrane contactor technology affords great opportunities for nitrogen recovery from waste streams. This study presents a performance comparison between lab- and pilot-scale membrane contactors using landfill leachate samples. Polypropylene (PP) and polytetrafluoroethylene (PTFE) fibers in different dimensions were compared in terms of ammonia (NH_3_) recovery on a lab scale using a synthetic ammonium solution. The effect of pre-treating the leachate with tannin coagulation on nitrogen recovery was also evaluated. An ammonia transfer on the lab and pilot scale was scrutinized using landfill leachate as a feed solution. It was found that PTFE fibers performed better than PP fibers. Among PTFE fibers, the most porous one (denoted as M1) had the highest NH_3_ flux of 19.2 g/m^2^.h. Tannin pre-treatment reduced fouling and increased NH_3_, which in turn improved nitrogen recovery. The mass transfer coefficient of the lab-scale reactor was more than double that of the pilot reactor (1.80 × 10^−7^ m/s vs. 4.45 × 10^−7^ m/s). This was likely attributed to the difference in reactor design. An analysis of the membrane surface showed that the landfill leachate caused a combination of inorganic and organic fouling. Cleaning with UV and 0.01 M H_2_O_2_ was capable of removing the fouling completely and restoring the membrane characteristics.

## 1. Introduction

Nowadays, fertilizers are needed in agriculture because the availability of nutrients in the soil is not enough to secure the increasing global food demand. Based on a report by the Food and Agriculture Organization of the United Nations (FAO), the fertilizer consumption on a global scale in 2020 was expected to be around 300 million tons and will keep increasing in the following years due to the growing population [1]. Nitrogen and phosphorus are the main components used for fertilizer production, but most fertilizers produced are based on nitrogen (N) [2]. The traditional way to produce fertilizers consists of fixing non-reactive nitrogen (N_2_) from the atmosphere to produce ammonia (NH_3_), which is also known as the Haber–Bosh process. The main drawback of this process is the high energy consumption (almost 1% of the world’s energy production) due to the high working temperatures and pressures [3]. The high energy consumption and environmental footprint of the nutrient removal from wastewater are other issues that have encouraged researchers and professionals to spend efforts on developing recovery technologies [4]. Indeed, nitrogen is removed in wastewater treatment plants (WWTPs) by the conversion of reactive nitrogen produced by human metabolic activities to non-reactive nitrogen, to be released back into the atmosphere [5]. This process is called nitrification/denitrification, and it is reported to consume up to 70% of WWTP energy [6]. Moreover, the contribution of this process to GHG emissions is reported to be between 0.1 and 0.9 kg of CO_2_ per cubic meter of wastewater treated [4,7]. Landfill leachate is one of the wastewater streams that is normally sent to WWTPs for treatment. This stream has a high nitrogen content that may negatively impact the biological processes if received in big portions [8]. Therefore, recovering nitrogen as ammonia directly from the waste stream or at the WWTPs could improve energy usage and mitigate the environmental impact in addition to producing potential sustainable fertilizers.

Various nitrogen and phosphorous recovery technologies have been developed and reported in the literature, even though only a few of them have been implemented on an industrial scale. For example, the SMART-plant project applies struvite precipitation and ion exchange to recover nutrients [9]. A full-scale stripper reactor coupled with a CO_2_ pre-stripper was installed and tested in 2010 at the Kloten/Opfikon WWTP in Switzerland. The wastewater stream was a mix of liquid fractions from an anaerobic digester and separate collected urine. This latter stream was treated beforehand by adding MgO to produce struvite, which was able to remove more than 95% of the phosphorus [10]. Another project developed by Helsinki Region Environmental Services (HSY), called the RAVITA project, tested a pilot plant based on phosphorus precipitation and recovery between 2016 and 2018 at the Viikinmäki WWTP. They also proposed the addition of an ammonia-stripping step prior to the phosphorus recovery, but this unit has not been realized yet [11]. It is worth mentioning, though, that HSY is currently planning a full-scale installation with local WWTPs. Other than existing projects on the pilot scale, many lab-scale experiments have been conducted to develop a variety of methods for nutrient recovery. These methods include the air stripping of ammonia, struvite precipitation, ion exchange, biological methods (biological assimilation), and membrane processes [4,10,12,13,14,15,16]. The progression pace of these methods varies significantly. Some of them, such as stripping, have reached a high technical maturity level, while others are still at the phase of feasibility evaluation. Among the developing technologies, membrane contactors seem to have great potential to reach the desired goal of recovering nutrients with low energy demands, as they operate at low pressures and temperatures.

Gas-permeable membrane technology is widely used in gas treatment applications [17,18]. However, in recent years, the use of membranes in wastewater treatment applications has gained considerable and polarized attention [13,15,19]. Membrane contactor technology has been tested for the recovery of ammonia nitrogen from wastewater streams. The driving forces of this process are the difference in partial vapor pressure and the concentration of the target compounds between the wastewater side and the absorbing-solution side (usually acid) [14]. The hydrophobic membrane does not allow water to pass through due to the surface tension effect; therefore, its pores are filled with air, allowing only gaseous compounds (such as ammonia) to diffuse through the pores [20]. Ammonia is then absorbed in the acid to form ammonium salts, which can be used in different industrial applications [21]. The advantages of this technology compared to the traditional strippers currently used are the lower energy required, the high selectivity toward ammonia, and the ability to reduce ammonia nitrogen to low levels [22]. The ammonia transfer rate is commonly used to characterize the efficiency of the process, and it is highly dependent on parameters such as the membrane configuration, pH, temperature, feed flow rate, material used, and composition of the feed [23,24].

Previous studies that have explored the application of membrane contactors for nitrogen recovery from landfill leachates have relied mostly on the use of commercial compact membrane contactors. These contactors are efficient at nitrogen recovery, but they require feed pre-treatment for the removal of solids to prevent blockages [25]. There are only a few examples in the literature that have reported results on membrane contactor pilot demonstrations, such as the study conducted by Haiqing et al. [26]. Our study investigated the nitrogen recovery from landfill leachate collected from the Ämmässuo site in Finland using membrane contactor technology at the lab and pilot scales. Our research group developed a membrane contactor pilot unit with a high solid tolerance (>500 mg/L) for the simultaneous recovery of N and P using PTFE fibers and ballasted sedimentation (called NPHarvest technology) [27]. The performance comparison between the lab and pilot scales can elucidate the effects of the scalability and reactor design on the recovery efficiency. The effect of the membrane fiber materials and characteristics were first investigated using a lab-scale reactor and a synthetic ammonium solution. This step helped to identify the best fiber characteristics for further system development. The impact of treating landfill leachate with tannins as a natural coagulant on the ammonia recovery was then investigated using a lab-scale reactor. Tannins were used to explore their dual effects of removing solids and organic carbons and increasing the ammonia concentration [28] on the recovery efficiency. Finally, fouling development and cleaning with H_2_O_2_ and UV were studied at the lab and pilot scales. The impact of fouling on the membrane surface was characterized using scanning electron microscopy and energy-dispersive X-ray spectroscopy (SEM-EDS), atomic force microscopy (AFM), contact angle (CA) measurements, and a Fourier-transform infrared spectroscopy (FTIR) analysis.

## 2. Materials and Methods

### 2.1. Feed Properties

There were three feed types tested in this study for addressing three research questions. A synthetic ammonium solution was used for testing the effect of membrane materials and characteristics on nitrogen recovery. Landfill leachate was applied at the lab and pilot scales for evaluating the developed membrane contactor capacity to recover nitrogen from the waste stream. The treatment of landfill leachate with tannins was employed for assessing the treatment effect on the nitrogen recovery and membrane fouling in a lab-scale reactor. The synthetic ammonium solution was prepared by dissolving NH_4_Cl in water to achieve an NH_3_-N concentration of 1000 mg/L. The landfill leachate samples were collected from the Ämmässuo site. The site is located in the city of Espoo (Finland) and it is one of the largest landfills in northern Europe. It is owned by HSY and it is divided into an old landfill area, which operated from 1987 to 2007, and a new landfill area, which was in active use from 2007 to April 2014. In the old landfill, approximately 10 million tons of waste were disposed during its years of operation. This landfill collected and disposed waste from the Helsinki metropolitan area, which covers around 1 million residents. A map of the landfill site with the sampling point highlighted is provided in Figure S1. This point was selected because of its higher NH_3_-N content compared to other points. The landfill leachate characteristics of the selected collection point are reported in Table 1.

### 2.2. Experimental Lab Set-Up

A lab reactor was built using an acrylic tube with a diameter of 0.075 m and a height of 0.15 m. Membrane fibers were installed inside the reactor with a connection to the acid line. The specifications for the different membrane fibers used (M1–M4) are provided in Table S1. The PTFE fibers were provided by Zeus^®^, and the PP bundle was purchased from Zena membranes. Considering the volume occupied by the different membrane fibers, the available reactor volume for the feed stream was 0.66 L, 0.62 L, 0.64 L, and 0.56 L for M1, M2, M3, and M4, respectively. This corresponded to area-to-volume (A/V) ratios of 18.0 m^−1^, 20.8 m^−1^, 12.6 m^−1^, and 339.0 m^−1^, respectively.

**Figure 1:**
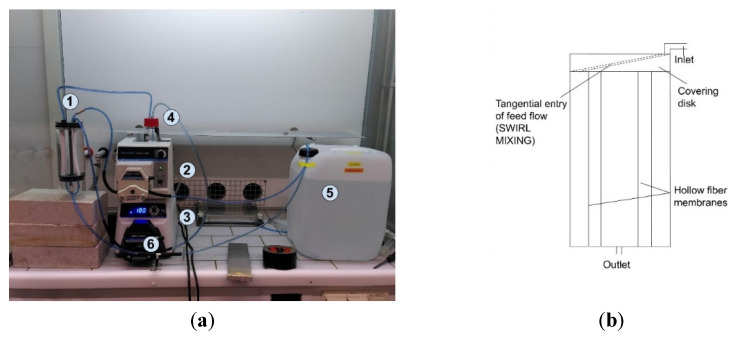
(**a**) Experimental set-up: (1) membrane reactor, (2) peristaltic pump for feed-stream side, (3) Masterflex peristaltic pump for acid side, (4) 80 mL acid container and sampling point for acid side, (5) 20 L tank for feed stream, and (6) T-valve used as sampling point for feed-stream side. (**b**) Schematic representation of the induced mixing mechanism.

Figure 1a shows the experimental set-up, consisting of (1) a reactor equipped with an inlet pipe on top, into which the feed stream was pumped from the 20 L feed tank (5) using a peristaltic pump (Masterflex) (2). An outlet pipe was placed at the bottom of the reactor, where the ammonia solution was pushed out due to hydraulic pressure. Another peristaltic pump (3) circulated the acid from and into the acid container (4). The amount of acid used for each experiment was 150 mL. In this study, the feed was circulated on the shell side while the receiving solution was circulated on the lumen side of the membrane. Samples were taken from the acid container, (4) and for the feed side, from a T-valve (6) placed on the outlet pipe. The hydraulic retention time (HRT) of the process was selected to be 8 h for consistency with the pilot reactor runs (corresponding to a feed flow of 20 L/h), and sulfuric acid was used as recommended by Kaljunen [29]. Owing to the designed tangential entry of the inlet, swirl mixing was induced in the reactor, as shown in Figure 1b.

### 2.3. Experimental Lab Procedure

As explained in Section 2.1, the experiments that were conducted with the lab-scale reactor involved using a synthetic NH_4_ solution with a concentration of 1000 mg/L, untreated landfill leachate, and treated landfill leachate with tannin coagulation. The membrane fibers M1–M4 were used with the synthetic NH_4_ solution for 24 h runs to study the impact of membrane characteristics on nitrogen recovery. Samples were collected during the tests from the acid and feed sides for ammonia concentration measurements. The M1 fiber was used for testing nitrogen recovery from untreated and treated landfill leachates. Details of the landfill leachate treatment with tannin coagulation have been provided in our previous work [28]. The pH of all feed solutions was raised to 11 using a 10 M NaOH solution to induce the conversion of ammonium to gaseous ammonia [13,25]. The effect of fouling formation on the nitrogen recovery from untreated and treated landfill leachates was also explored using an extended run time of 72 h. Fouled membrane fibers were cleaned with UV and 0.01 M H_2_O_2_ (provided from VWR) for 1 h. Then, the cleaned membrane was tested for nitrogen recovery using treated landfill leachate as a feed solution.

### 2.4. Pilot-Scale System Description

**Figure 2:**
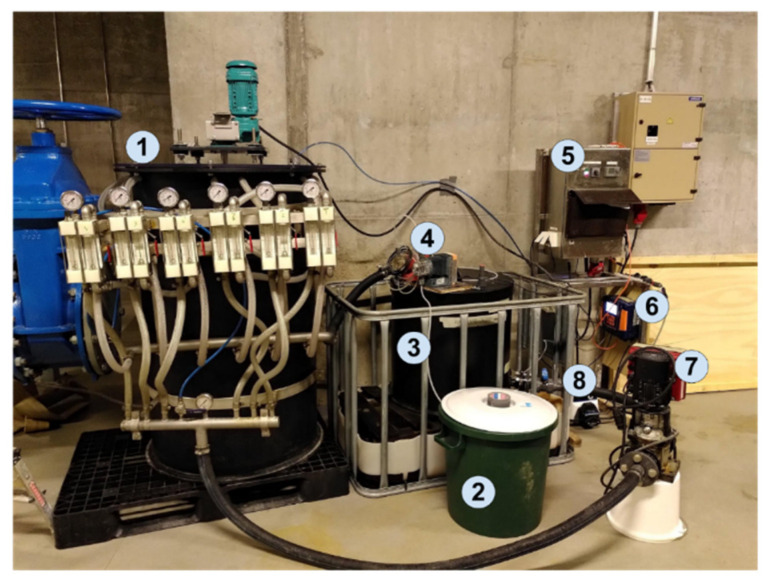
Ämmässuo pilot test set up: (1) membrane reactor, (2) NaOH container, (3) H_2_SO_4_ container, (4) NaOH pump, (5) power station, (6) controller DULCOMETER diaLog DACb, (7) H_2_SO_4_ pump, and (8) peristaltic pump (Masterflex) controlling the feed flow rate.

The pilot system is shown in Figure 2. A peristaltic pump (Masterflex) (8) was placed between the source point and the reactor (1) to adjust the flow rate according to the selected hydraulic retention time (HRT). In this test, the flow rate was 40 L/h, and it was set as such to obtain an 8 h HRT. Before the leachate entered the reactor, its pH was adjusted using a 32% NaOH solution stored in a 40 L tank (2). The controller DULCOMETER diaLog DACb (6) constantly measured the pH from the inlet and was connected to the NaOH pump (4), so that the alkaline solution was automatically dosed to ensure that the pH was over 10 during the process. The acid was pumped from the acid tank (3) to the reactor (1) with a second pump (7). The pumps, pH control unit, and motor of the mixer were controlled by a programable logic controller (5).

The tank contained 6 membrane fiber modules. Each module contained 100 fibers of the M1 membrane. The liquid inside the reactor was mixed using a hyperbolic mixer. The acid volume used was 140 L. The membrane fibers took up to 100 L when they were filled. As shown in Figure 2, each module had a designated valve and pressure gauge to control the acid flow individually if need be. In the case of the study, all modules operated on the same flow level of 5 L/h per module, which produced a collective acid flow through the six modules of 30 L/h.

### 2.5. Pilot Test Procedure

The pilot tests were conducted for three days. During this time, samples were collected in duplicate and stored in the fridge until the time of analysis. Four different sampling points were selected in order to keep track of the ammonia concentration throughout the process, as listed below.

- Inlet: after the pH adjustment and before entering the reactor;
- Bulk: inside the reactor, at around a 0.5 m depth from the top;
- Outlet: after the treatment and before discharging into the sewer;
- Acid: taken from the acid side.

### 2.6. Analysis

Samples collected during the experiments were stored in the fridge prior to analysis, and were then analyzed for ammonia concentration by applying APHA 4500-NH3 D (ammonia-selective electrode method) using an Orion ammonia probe, model 290A. After the completion of the experiments and pilot tests, samples of the membrane fibers were taken for studying the fouling accumulation and cleaning method efficiency by applying a range of surface characterization techniques. The surface morphology and elemental composition were analyzed using SEM-EDS (JSM—7500FA, JEOL) with an accelerating voltage of 10 kV and a probe current of 10 pA. The membrane samples were coated with a 5 nm thickness of Au–Pd prior to the SEM-EDS analysis. The topography of the membrane samples was obtained using a Nanoscope 1.5 MultiMode 8 AFM (Bruker) with the tapping mode in air, and the roughness was analyzed with the NanoScope Analysis 1.5 software (Bruker). The CA of the membrane samples was measured with a Theta Flex optical tensiometer (Biolin Scientific) and calculated with the Young–Laplace equation. The CA was measured by dropping deionized water onto the membrane surface with a 300 μL automatic pipe (sessile drop method). The size of the drop was 4 μL. The chemical structure of the membranes and the fouling layer were investigated using FTIR-ATR (PerkinElmer).

## 3. Results and Discussion

### 3.1. Effect of Membrane Characterization on NH_3_ Recovery and Transfer Rate

**Figure 3:**
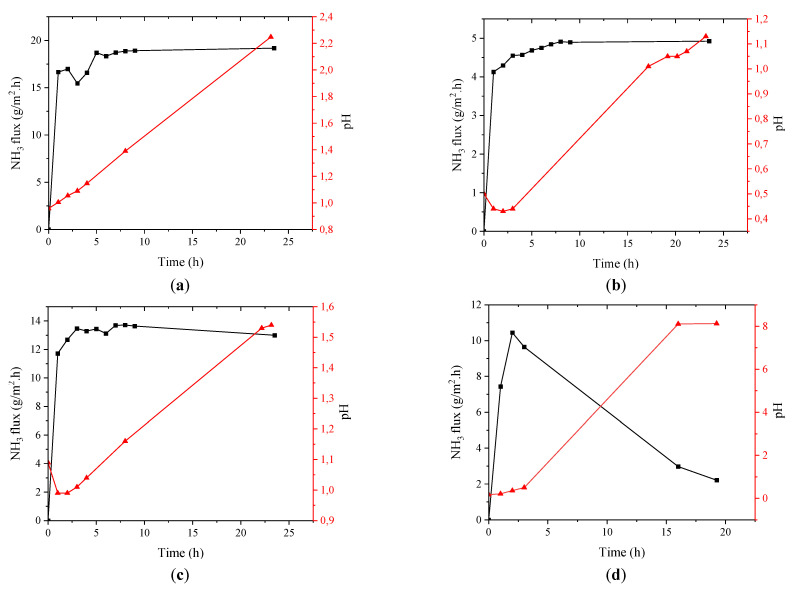
Ammonia flux and acid pH of (**a**) M1, (**b**) M2, (**c**) M3, and (**d**) M4.

This section presents and discusses the results of testing M1–M4 for recovering ammonia from a synthetic ammonium solution. Prior to testing the different fibers, a short experiment of 4 h for testing the mixing effect on NH_3_ recovery was conducted using M1, and the results are shown in Figure S2. It was clear that mixing had a significant effect on the recovery, and hence, the rest of the experiments were conducted with mixing. Figure 3 shows the NH_3_ flux across the tested fibers, along with the accompanying change in acid pH. The flux was calculated based on the accumulated ammonia in the acid. It can be seen that M1 had the highest NH_3_ flux, followed by M3 and M4, while M2 had the lowest flux. The pH of the acid for the ePTFE membrane followed the same trend and reached a maximum of 2.3. The case was different for the PP membrane, as the pH shot up to 8 around the time mark of 17.5 h. The acid volume was also noticed to double after the 24 h test. Such a high increase in acid volume was not observed with the ePTFE membranes. This indicates acid exhaustion and the leaching of water across the PP membrane.

**Figure 4:**
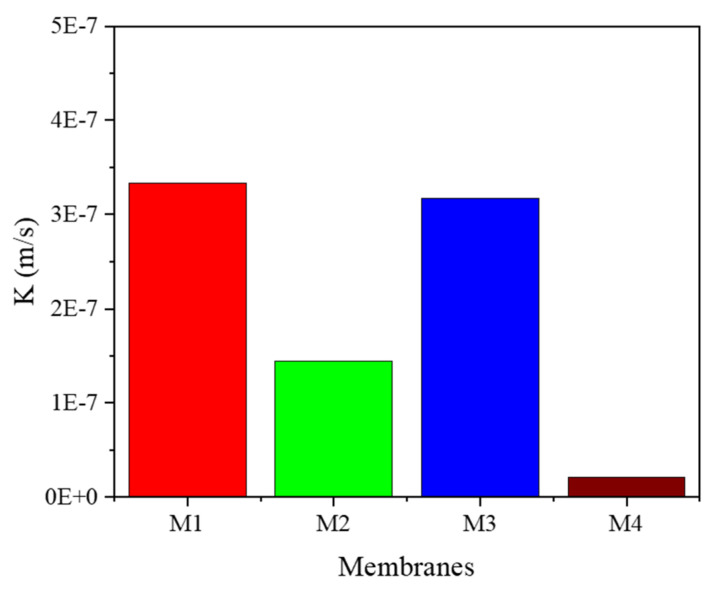
Mass transfer coefficient of tested membrane fibers using a synthetic ammonia solution.

The ePTFE membranes with a wall thickness of ≤100 µm (M1 and M3) had a higher flux than the PP membrane; however, the ePTFE membranes with a wall thickness of >100 µm had a lower flux compared to the PP membrane. When comparing the properties of the ePTFE membranes (M1–M3), it seems that porosity played a bigger role in the NH_3_ transfer rate compared to the membrane thickness. Despite M3 having a thinner wall compared to M1, the NH_3_ flux was still higher for the latter as opposed to the former. This was likely due to the high porosity range of M1 (70–90%) compared to M3 (50–70%). Based on the results presented in Figure 1, it appears that the pore size did not have an effect on the NH_3_ transfer rate, as all ePTFE membranes had the same pore size, yet they had significantly different fluxes. Some of the conclusions drawn from this work are in agreement with those reported by Lauterböck and co-workers [30], who studied the impact of membrane characteristics on ammonia transfer for commonly used materials in recovery applications. Although their statistical model suggested a negative correlation between ammonia transfer and thickness, it contradicted with the results of other studies [30] regarding a suggested negative correlation between porosity and the ammonia transfer rate. A notation made by the Lauterböck team that is worth discussing here is the tradeoff between small pores that impose high resistance against the flow, but at the same time are less prone to wetting; the opposite is true for large pores. The observations of this study suggest that porosity is more important than pore size when it comes to ammonia transfer; however, our results were based on the use of a synthetic solution, so fouling was not considered. In general, the ePTFE membrane used in our studies (current and previous [27]) has shown to be resistant to wettability and was able to maintain almost the same contact angle throughout the testing (see Table 2). The other membrane characteristic that can influence gas diffusion is hydrophobicity. The CA of a PP membrane similar to the one used in this study was reported to be 102° [31], which is less than that of the ePTFE membrane (>120°, Table 2) measured in this study. This could be another reason for the superior performance of PTFE compared to PP. Tortuosity can also affect gas diffusion through membrane pores (see Equation (1)) [32]. The tortuosity of the ePTFE membrane was calculated applying Equation 2 [33], and the results are presented in Table S1. The membrane with the lowest tortuosity had the highest flux. All in all, the porosity seems to be the most important factor to consider when selecting membranes for designing contactors for ammonia recovery. Thickness is also important, especially when considering a high fluid flow rate on the lumen side. The mass transfer coefficients of the different fibers were calculated by applying Equation (3) [27], and the results are provided in Figure 4. M1 had the highest coefficient, followed by M3, then M2, and finally M4. The obtained *K* values were in the same range as those reported for the PTFE and PP membranes in [30]. However, the PTFE *K* values were lower than those found for synthetic digestate [34], and the reason behind this could be the difference in membrane thickness. Our membranes were about 3–10 times thicker than theirs. The *K* value for the PP membrane obtained in this study was also lower than those calculated for a synthetic urine solution, but again, the dimensions of the membrane fibers differed from those of our fibers. Direct comparisons with literature values can be very challenging, as there is only a slim chance of finding identical materials and testing conditions to our study.

(1) $$K=\frac{D\times \varepsilon }{T\times \tau }$$

(2) $$\tau =\frac{{\left(2-\varepsilon \right)}^{2}}{\varepsilon }$$

(3) $$\ln\frac{C_{{}^{\circ}}}{C}=\frac{KA}{V}\;t$$

In the above equations, *K* is the overall mass transfer coefficient of NH_3_ (m/s), D is the NH_3_ diffusion coefficient in the pores (m^2^/s), ε is the membrane porosity (%), τ is the membrane tortuosity (-), T is the membrane thickness (m), C_0_ is the initial concentration of NH_3_ in the liquid (mg/L), C is the NH_3_ concentration in the liquid at time t (mg/L), A is the membrane surface area (m^2^), V is the reactor volume (m^3^), and t is time (s).

### 3.2. Ammonia Recovery from Landfill Leachate: Lab Scale vs. Pilot Scale

**Figure 5:**
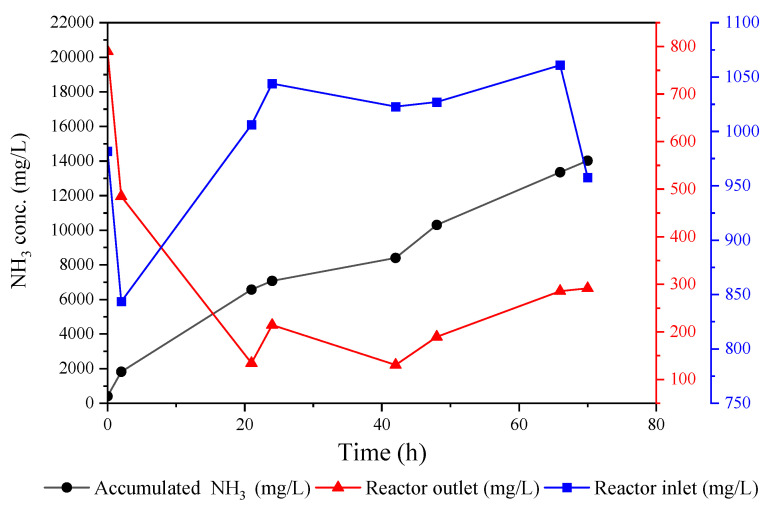
Ammonia concentration profile for contactor and acid tanks throughout the testing period.

The ammonia concentration profile for the inlet, outlet, and acid tank of the pilot reactor is shown in Figure 5. The fluctuations in the inlet concentration reflect the variation in the ammonia concentrations of the produced leachate at the Ämmässuo site, as the reactor was connected directly to the source point of the collected leachate without a pre-treatment step. It can be seen that the reactor was efficient at recovering ammonia from the fed leachate. The NH_3_ removal was, on average, 67%, and in some instances was recorded to be as high as 87%. The final ammonium sulfate concentration reached 17 g/L, and the increase in the accumulation curve’s sharpness after 40 h clearly indicated that the acid still had the capacity to absorb more ammonia. Despite the fact that our system treated high-carbon-content raw leachate without pre-treatment, it still produced comparable results to studies conducted with pre-filtered leachate with a lower carbon content, such as the study conducted by Amaral et al. [25]. This study reported an NH_3_ recovery between 13.6% and 100%, and their TOC concentration was considerably lower than that of the Ämmässuo leachate (412 mg/L vs. 1189 mg/L). In addition, our system was not completely sealed, so there was a possibility of ammonia loss to the atmosphere. However, this issue has been improved in the recent version of our pilot unit and further developments are still underway.

**Figure 6:**
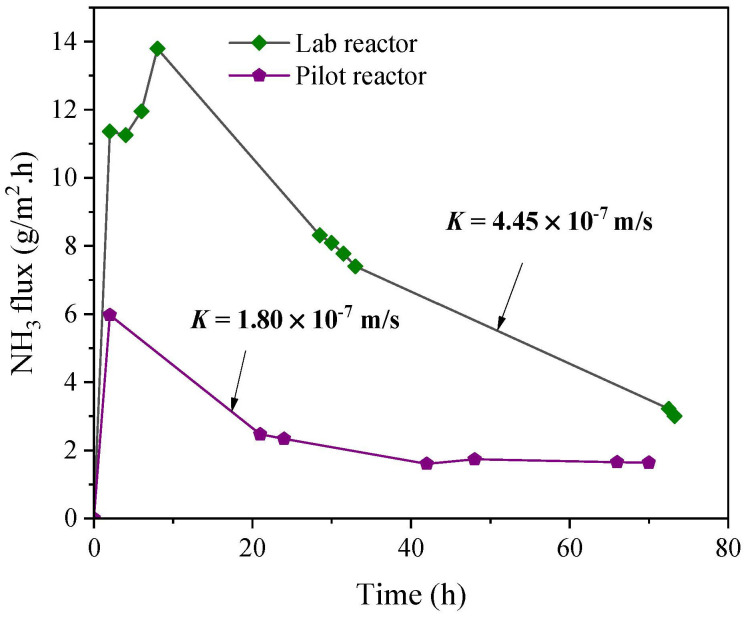
NH_3_ flux of lab- and pilot-scale reactors using untreated landfill leachate.

The ammonia recovery from untreated landfill leachate was also tested at a lab scale for a 72 h run. The obtained flux from this test has been plotted together with the flux recorded from the pilot trials in Figure 6. It was clear that the lab reactor had a better ammonia transfer rate compared to the pilot reactor, as marked by a higher flux and mass transfer coefficient. The reason behind this difference in the performance is believed to be due to the reactor design and packing density, as the flow and other parameters were set to be the same for both reactors. The major design difference was in the mixing mechanisms. A hyperbolic mixer was used in the pilot-scale reactor, while induced swirling was employed for mixing the liquid in the lab-scale reactor. These aspects influenced the contact between the liquid and the membrane surface, as well as the liquid renewal in the adjacent area to the membrane. The calculated packing density of the pilot reactor was found to be 18.75 times higher than that of the lab reactor. The disadvantage of having a densely packed membrane module is the low mass transfer rate; however, this was offset by the high ammonia removal due to the availability of a large surface area. The ammonia removal with the lab reactor was about 5.5% compared to 67% for the pilot reactor. A good balance between the packing density and the ammonia transfer rate needs to be struck in order to achieve an efficient recovery process.

**Figure 7:**
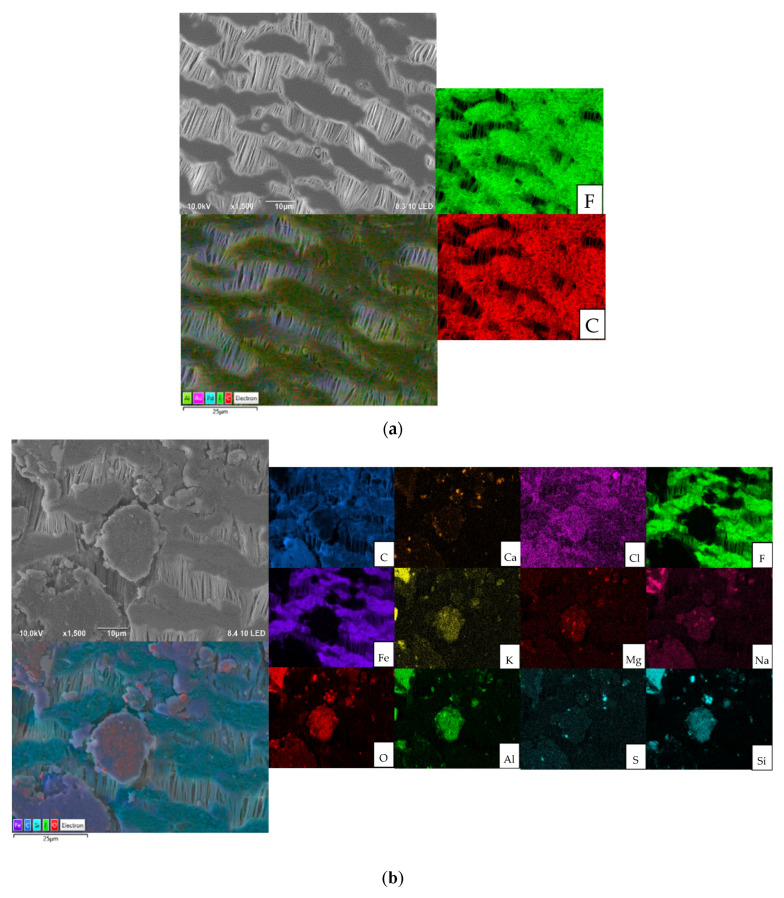

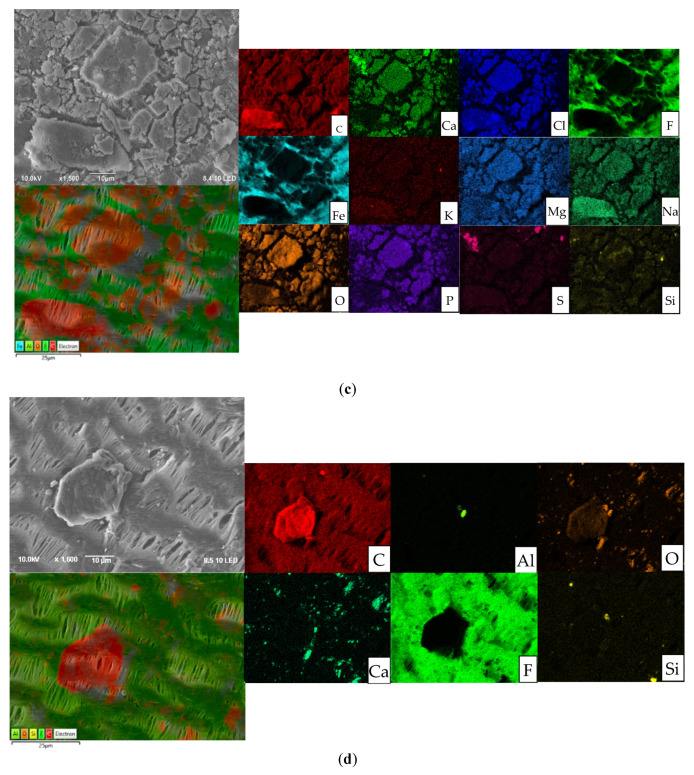

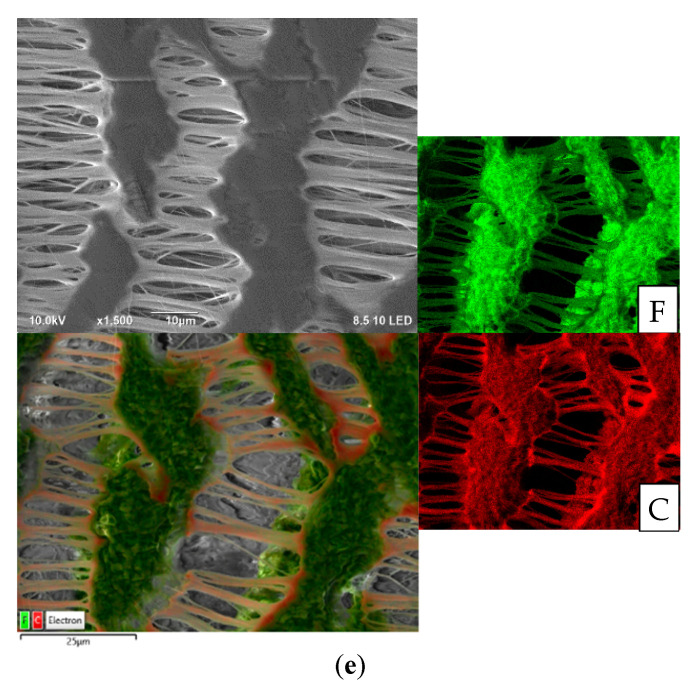
SEM-EDS maps for (**a**) virgin membrane, (**b**) fouled membrane from lab-scale experiment, (**c**) fouled membrane from pilot run, (**d**) fouled membrane of leachate treated with tannins, and (**e**) fouled membrane with untreated leachate after UV + H_2_O_2_ cleaning.

**Figure 8:**
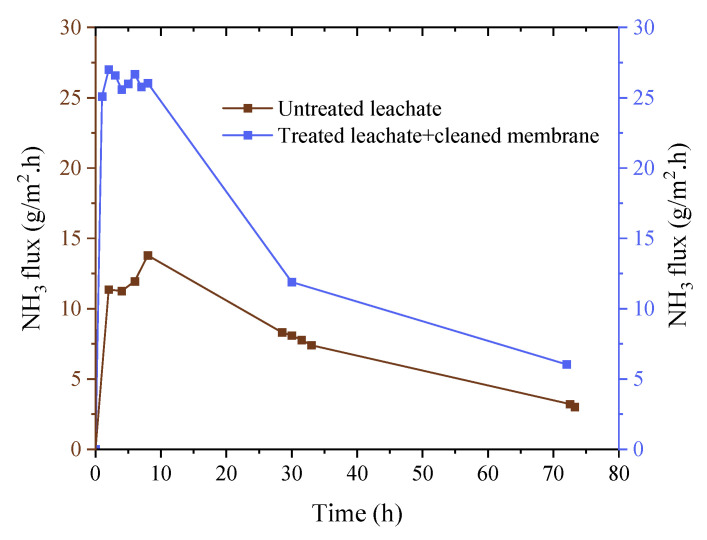
Ammonia flux of untreated leachate vs. treated leachate using a cleaned membrane with UV + H_2_O_2_.

**Figure 9:**
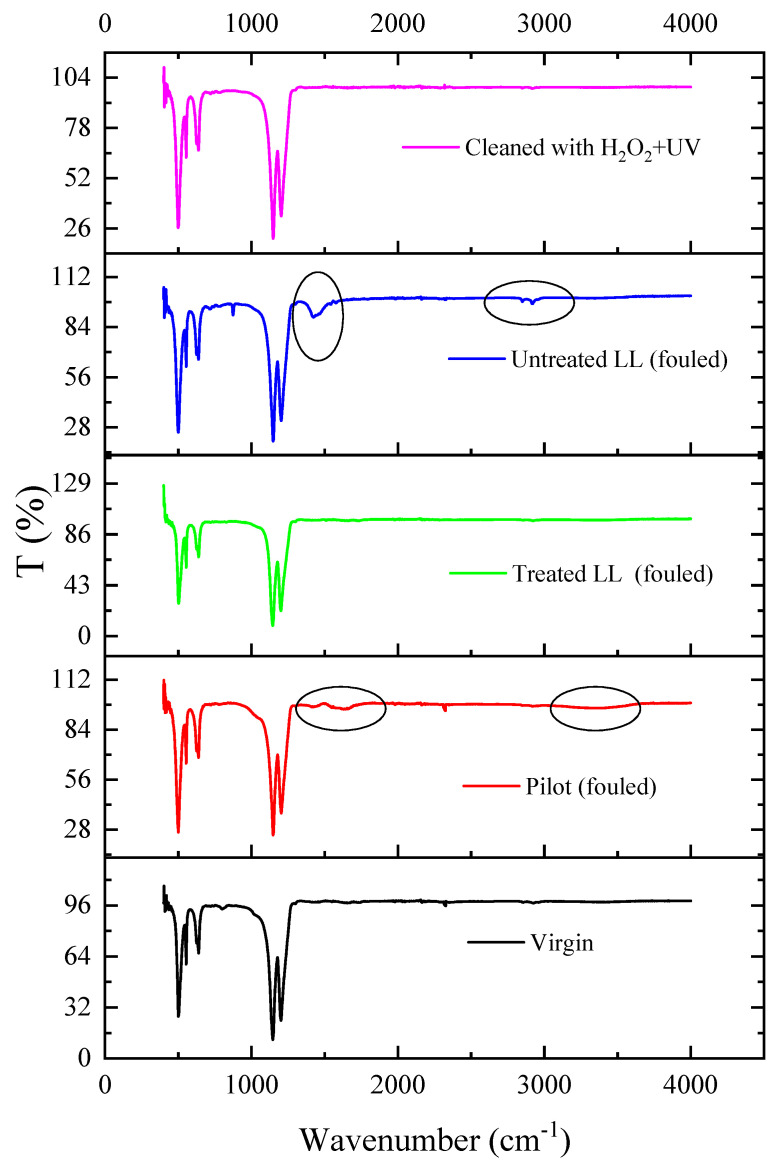
FTIR spectra of virgin, fouled, and cleaned membranes.

The impact of treating landfill leachate with tannin coagulation on the ammonia recovery was evaluated, and the recorded ammonia accumulation levels are shown in Figure S3. It was observed that treating the leachate with tannins improved the ammonia recovery. The accumulated ammonium sulfate wight increased from 24.9 g/L for the untreated leachate to 49.2 g/L for the treated leachate. This was attributed to the reduction of organic carbon (15% [28]) and other inorganic constituents (refer to Figure 7), which consequently led to a decrease in fouling formation and an increase in the ammonia concentration by ~18% after the tannin coagulation. The fouled membrane after a 72 h run with untreated Ämmässuo landfill leachate was cleaned with UV and H_2_O_2_, as described in Section 2.3, and re-tested with treated leachate for the same period of time. The ammonia flux of this test along with the flux of the untreated leachate are shown in Figure 8. The results presented in this figure and in Figure S3 show that cleaning with UV + H_2_O_2_ further improved the ammonia recovery when used with treated leachate. This suggests that cleaning with UV + H_2_O_2_ is capable of restoring membrane properties and slightly improving them further. The cleaning might have removed dirt already attached to the virgin membrane during manufacturing and shipping, as the intensity of the FTIR characteristic peaks of the cleaned membrane were slightly higher than their counterparts for the virgin membrane (Figure 9).

### 3.3. Fouling Development and Cleaning Effects

The surface morphology and chemistry were thoroughly investigated before and after the fouling and cleaning of the tested membranes. The SEM images and EDS maps of the membrane samples are presented in Figure 7. The virgin membrane sample showed a typical elemental composition of PTFE materials, consisting of F and C. The maps of the fouled membranes with the untreated leachate for both the lab and pilot reactors revealed that organic and inorganic foulants accumulated on the membrane surface during the tests. It can be seen that C and O followed the same pattern, suggesting the presence of a C-O structure, either in the form of organic or inorganic compounds. The similarity in the EDS map patterns for Na, Mg, Al, and Si with C and O indicate the existence of metal oxides and inorganic salts (e.g., MgCO_3_). There were other elements detected in the developed fouling layer from the untreated leachate, such as S, Fe, Cl, and P. The EDS elemental percentages of the membrane samples are provided in Figure S4. Recent studies have detected similar spectra for the elements in the landfill leachate foulant of PTFE membranes [35,36]. The use of tannin coagulation significantly dropped the metal content of the fouling layer, as can be seen in Figure 7d. However, carbonous fouling was still present, but to a lesser degree. The tannins were not as efficient at removing Ca, Si, and Al as the other elements. Cleaning the membrane with UV + H_2_O_2_ was efficient at removing all the elements except for traces of Al (see Figure S4e).

Further investigation into the chemical structure of the fouling layer was performed using an FTIR analysis of the membrane samples, as shown in Figure 9. The virgin membrane exhibited the typical four characteristic peaks of PTFE. The peaks around 1150 cm^−1^ and 1200 cm^−1^ were associated with the vibration of the -CF_2_ and -CF_3_ groups [37]. The peaks at 500 cm^−1^, 550 cm^−1^, and 650 cm^−1^ were related to the bending modes of the -CF_2_ group [38]. The reduction in the intensity of these peaks suggested the accumulation of a fouling layer on the membrane, as is the case with fouled membranes. Some of the intensity of these peaks was restored with the treated leachate. However, the intensity of the characteristic peaks for the cleaned membrane were slightly higher compared to that for the virgin membrane. As explained earlier, this could have been due to the effective cleaning of UV + H_2_O_2_, which could have even removed particles adhered onto the virgin membrane surface during the manufacturing or shipping stages. The main peaks that appeared after fouling were a peak at the wavenumber of 3400 cm^−1^ and another at 1480 cm^−1^. The peak at 3400 cm^−1^ was believed to be linked to the presence of O-H intermolecular stretching from phenol, hydroxyl, and carboxyl functional groups [39]. The appearance of a peak at 1480 cm^−1^ was reported to be due to the presence of CH_2_ and CH_3_ aliphatic groups [37]. Some inorganic salts have also been reported to have characteristic peaks around these wavenumbers. For instance, CaSO_4_·2H_2_O has a characteristic peak at 3494 cm^−1^, and Na_2_CO_3_ has a characteristic peak at 1420 cm^−1^. The elements of these salts were present in the EDS maps of the fouled membrane, and this indicates that the landfill leachate might have caused organic and inorganic fouling.

**Figure 10:**
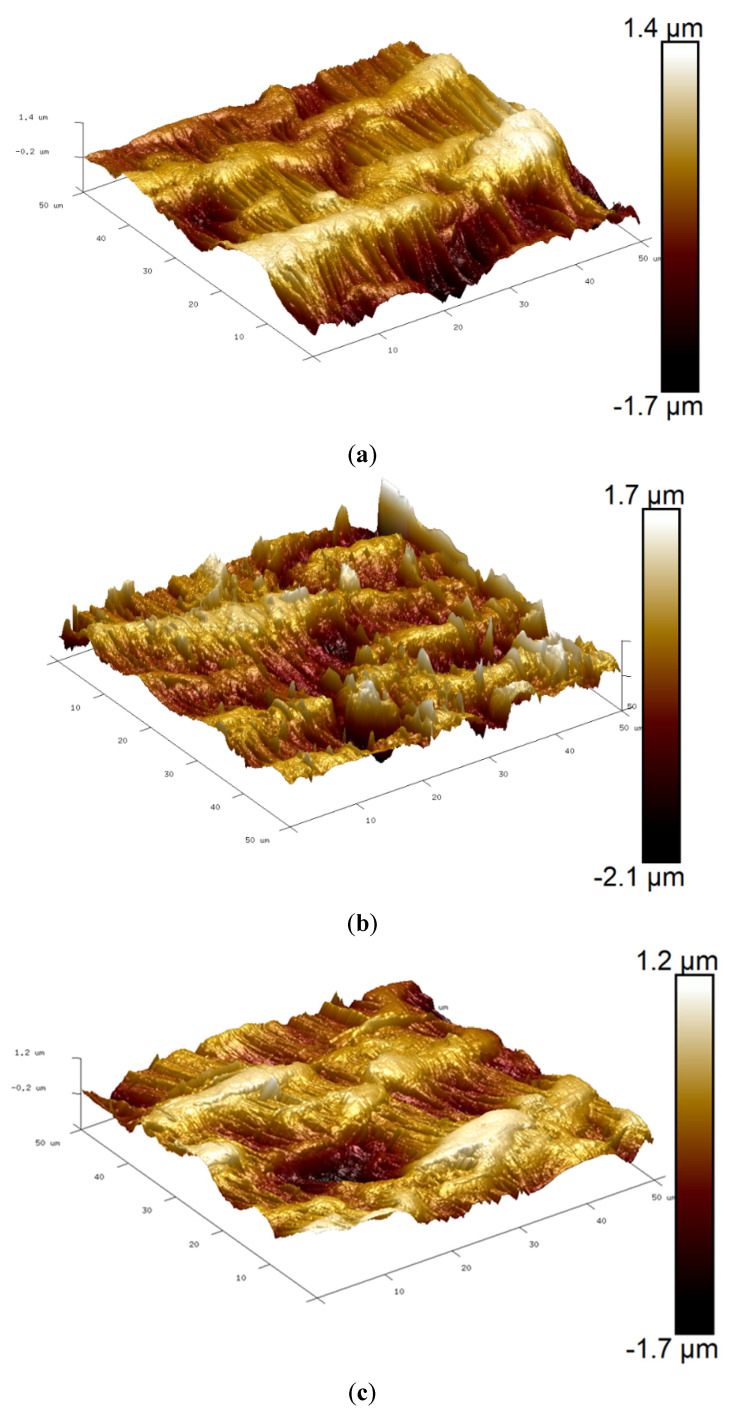

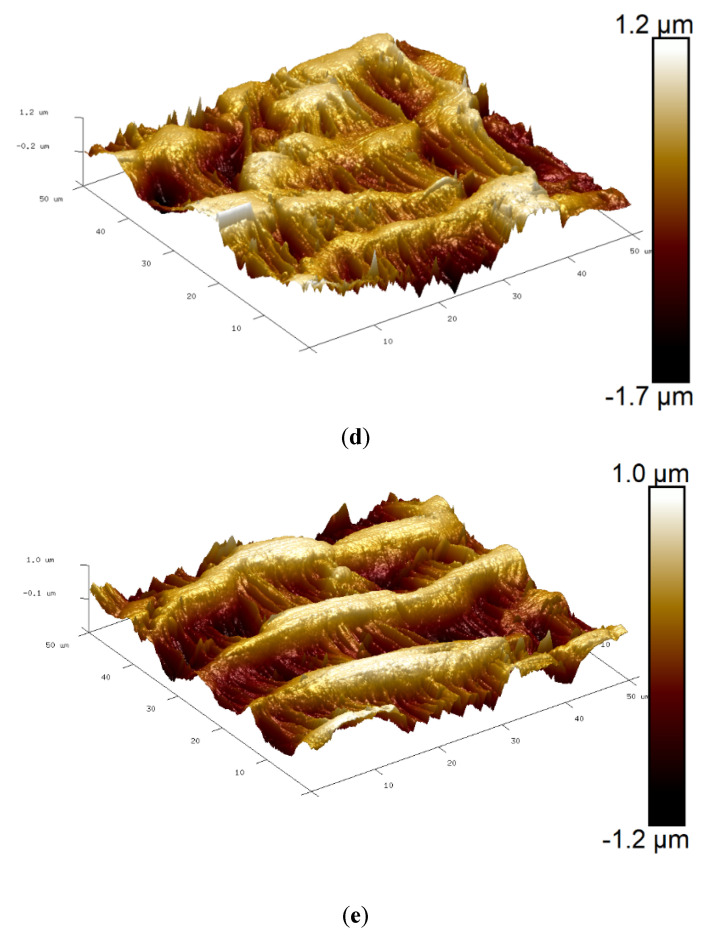
Three-dimensional AFM images of (**a**) virgin membrane, (**b**) fouled membrane from pilot run, (**c**) fouled membrane from lab test with untreated leachate, (**d**) fouled membrane from lab test with treated leachate, and (**e**) membrane cleaned with UV + H_2_O_2_.

Changes in membrane hydrophobicity and roughness were also examined, and the results are shown in Table 2 and Figure 10. It can be seen that fouling and cleaning did not significantly affect the membrane hydrophobicity, as the change in CA was only a few degrees. The change in roughness was rather noticeable, especially for the membrane sample from the pilot test. This was also clear in the 3D AFM photographs.

## 4. Conclusions

The nitrogen recovery from landfill leachate was studied in this work by using an in-house developed membrane contactor pilot unit and a small lab reactor. First, the performance of two commonly used membrane fibers, namely PTFE and PP, was tested using a synthetic ammonia solution. Three PTFE fibers with different characteristics were used. It was found that the membrane porosity had the highest effect on the nitrogen recovery. The PTFE with the highest porosity (M1) recovered the most nitrogen. The M1 membrane was tested for recovering nitrogen from untreated leachate using lab- and pilot-scale reactors. The latter achieved a decent ammonia removal of 67%, which was comparable to the removal reported by studies using commercial contactors with pre-filtered leachate. This reflects the high efficiency of our system (NPHarvest) for nitrogen recovery from landfill leachate. The lab reactor had a higher ammonia flux and mass transfer coefficient of more than double that of the pilot reactor, likely due to the significantly lower packing density and the difference in the design, which impacted ammonia flow and the interaction with the membrane surface. Treating leachate with tannin coagulation improved the ammonia recovery. The improved ammonia recovery was the result of a fouling reduction and an increase in the ammonia concentration of the treated leachate. Analyzing fouled membrane surfaces revealed that the leachate caused a combination of organic and inorganic fouling in the form of metal oxides and salts. The accumulated foulants on the membrane surface did not affect the membrane hydrophobicity, but it increased roughness, especially for the membrane sample from the pilot runs. Tannins were more efficient at removing organic fouling compared to inorganic fouling. Cleaning the membrane with 0.01 M H_2_O_2_ and UV was capable of restoring the membrane properties.

## Figures and Tables

**Table 1:** Characteristics of Ämmässuo leachate from Point T2.

Constituents	Concentration (mg/L)
Total nitrogen	1019.0
Total phosphorous	7.7
Total organic carbon	1189.0
Suspended solids	20.0
Volatile suspended solids	25.0
Ammonia	969.0
pH	7.9

**Table 2:** Contact angle and roughness parameters for virgin, fouled, and cleaned membrane samples.

Membrane Sample	Contact Angle (°)	Mean Roughness, Ra (nm)	Root Mean Square Roughness, Rq (nm)
Virgin	123 ± 0.07	360 ± 11	451 ± 15
Ämmässou pilot	121 ± 0.60	435 ± 18	557 ± 24
Ämmässou lab—untreated leachate	119 ± 0.3	383 ± 09	483 ± 10
Ämmässou lab—treated leachate	121 ± 0.05	361 ± 22	465 ± 15
Ämmässou lab—cleaned with UV + H_2_O_2_	120 ± 0.06	338 ± 13	418 ± 19

## Data Availability

Not applicable.

## Supplementary Materials

The following supporting information can be downloaded at: https://www.mdpi.com/article/10.3390/membranes12090837/s1, Table S1: Membrane fiber specifications based on manufacturer information. Figure S1: Ämmässuo landfill site and collection point T2. Figure S2: Effect of mixing on NH3 recovery. Figure S3: The effect of tannin coagulation and UV + H_2_O_2_ cleaning on ammonia recovery from landfill leachate. Figure S4: EDS elemental percentage of (a) virgin membrane, (b) fouled membrane from lab-scale experiment, (c) fouled membrane from pilot run, (d) fouled membrane of leachate treated with tannins, and (e) membrane cleaned with UV + H_2_O_2_.
