# Supplementary material for: Nitrogen Recovery from Landfill Leachate Using Lab- and Pilot-Scale Membrane Contactors: Research into Fouling Development and Membrane Characterization Effects
Source: Membranes (Basel). 2022 Aug 27;12(9):837. doi: 10.3390/membranes12090837 (PMC9503888; doi:10.3390/membranes12090837)
Supplement: Supplementary file 1 [file membranes-12-00837-s001.zip › membranes-1887467-supplementary.pdf]

Nitrogen recovery from landfill leachate using lab- and pilot-scale membrane contactors: Research into fouling development and membrane characterization effects

Ilaria Righetto[1,2], Raed A Al-Juboori[2], Juho Uzkurt Kaljunen[2], Ngoc Huynh[3], Anna Mikola[2]

1) Department of Environment, Land and Infrastructure Engineering, Politecnico di Torino, Corso Duca degli Abruzzi, 24, 10129 Torino, Italy
2) Water and Environmental Engineering Research Group, Department of Built Environment, Aalto University, P.O. Box 15200, Aalto, FI-00076, Espoo, Finland
3) Department of Bioproducts and Biosystems, School of Chemical Engineering, Aalto University, Espoo, Finland

Supplementary Materials

Table S1. Membrane fiber specifications based on manufacturer information.

**Membrane code**	Material	Internal diameter (cm)	Wall thickness (cm)	Number of fibers	Pore diameter (μm)	Porosity range (%)	Tortuosity (-)	Density (g/cm³)
**M1**	ePTFE	1.00	0.100	2	0.2-1.0	70-90	2.4-1.3	0.45±0.15
**M2**	ePTFE	1.11	0.127	2	0.2-1.0	50-70	4.5-2.4	0.85±0.15
**M3**	ePTFE	0.76	0.051	2	0.2-1.0	50-70	4.5-2.4	0.65±0.15
**M4**	PP	0.04	0.009	570	0.1	-	-	-

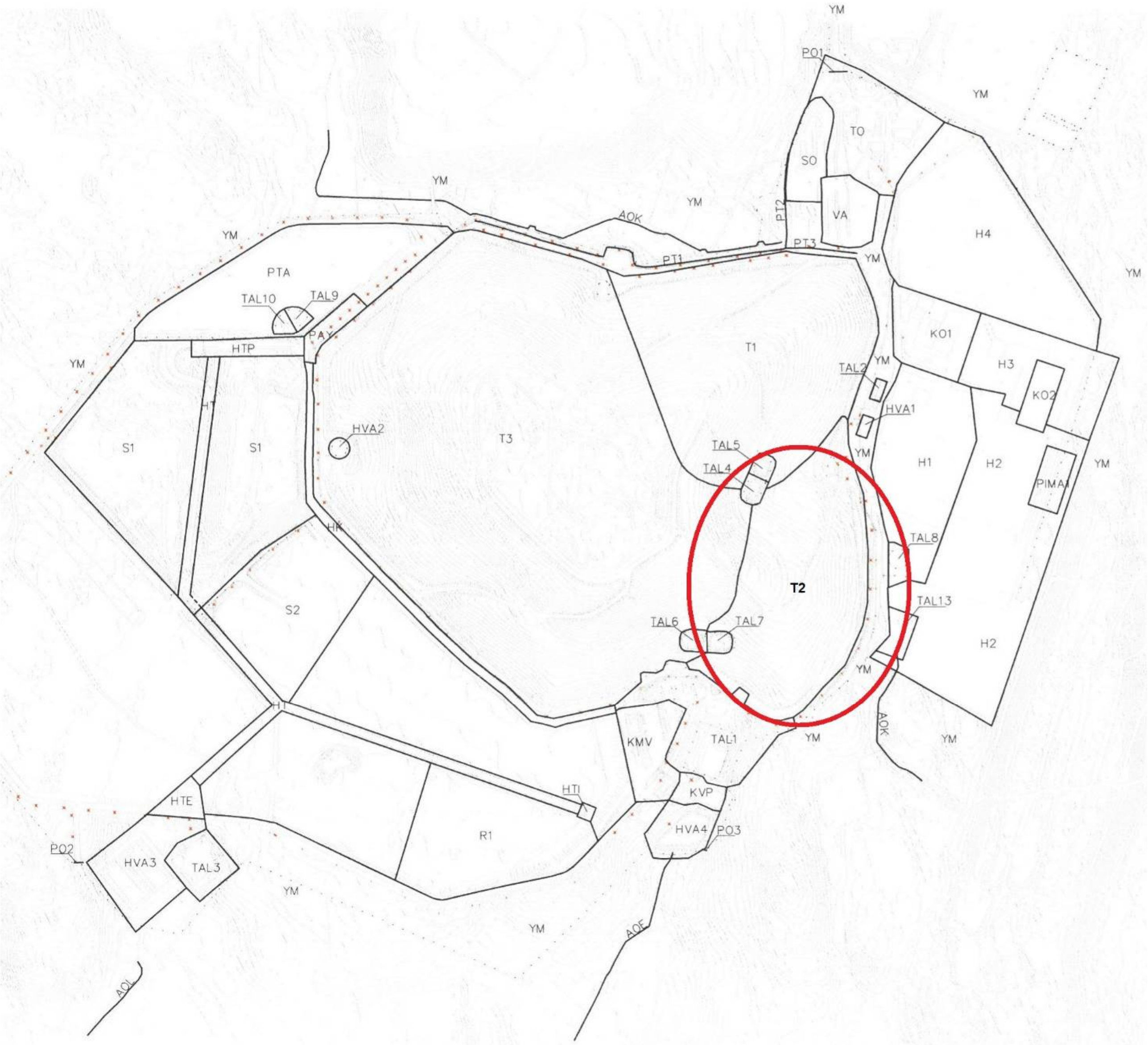

Figure S1. Ämmässuo landfill site and collection point T2.

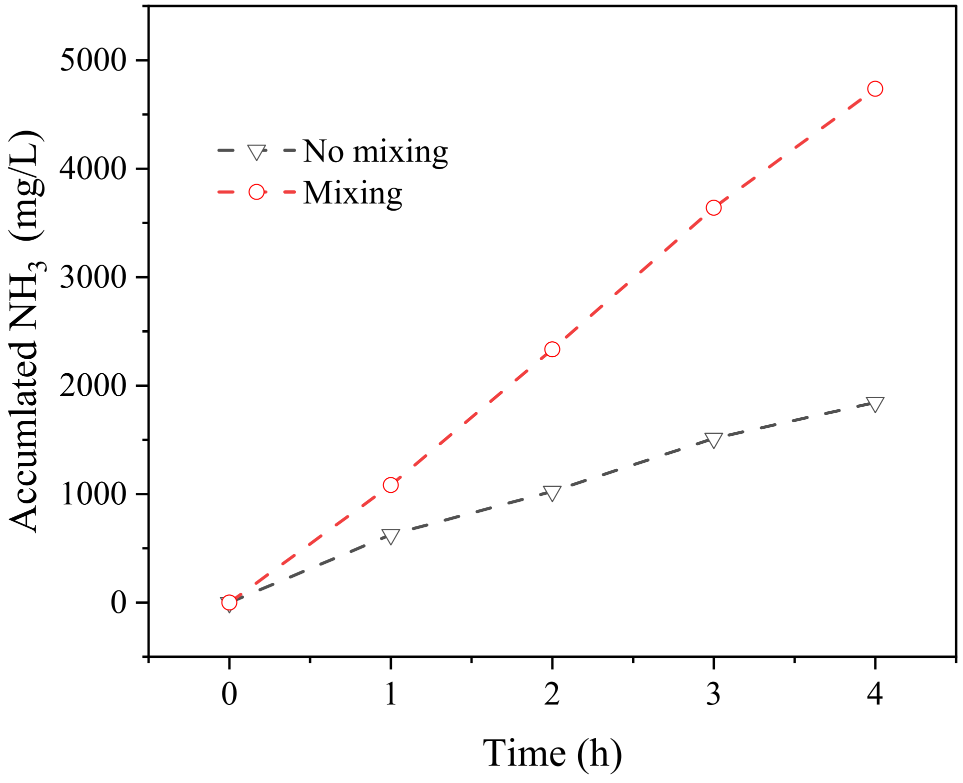

Figure S2. Effect of mixing on NH_3 recovery.

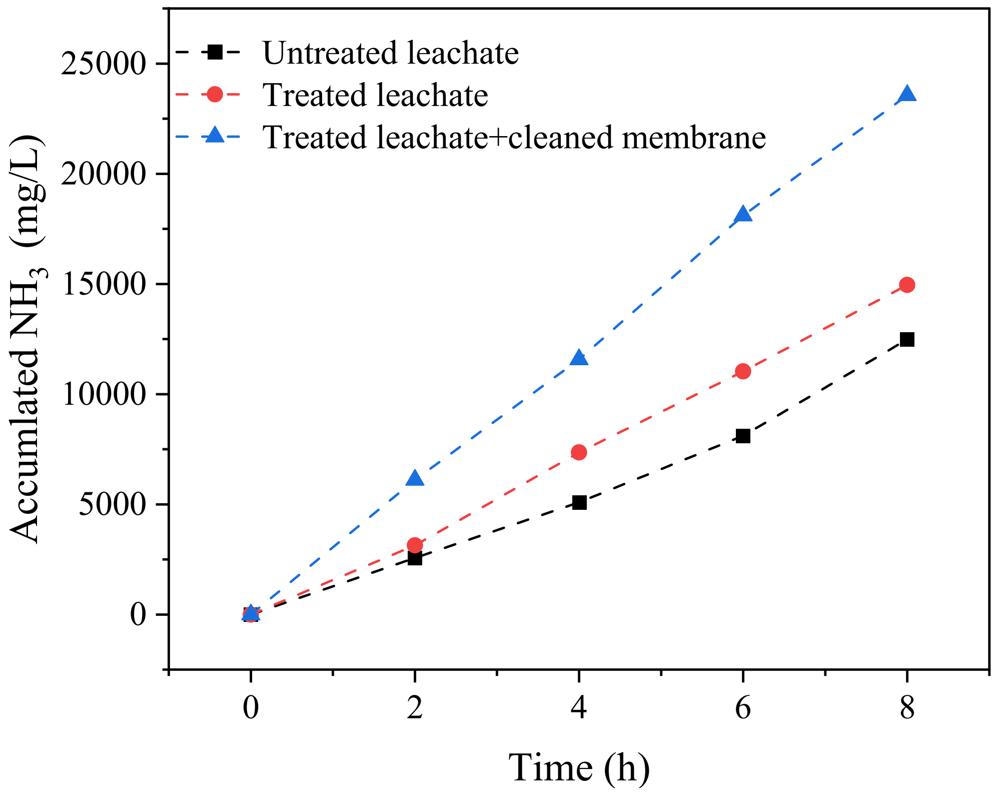

Figure S3. The effect of tannin coagulation and UV+H_2O_2 cleaning on ammonia recovery from landfill leachate.

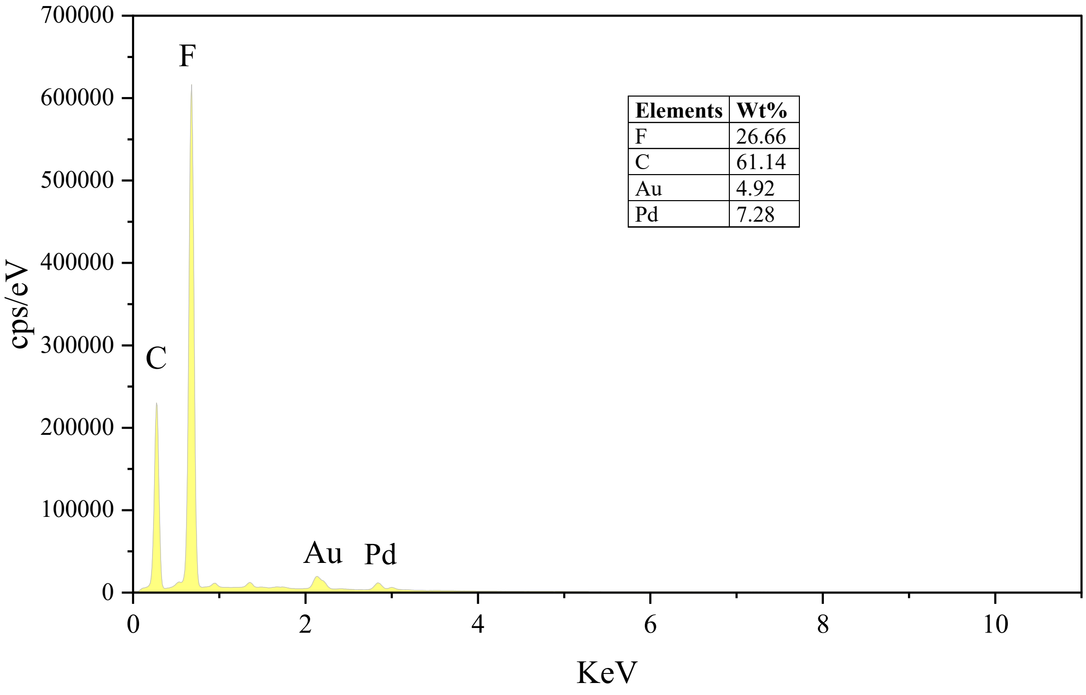

(a)

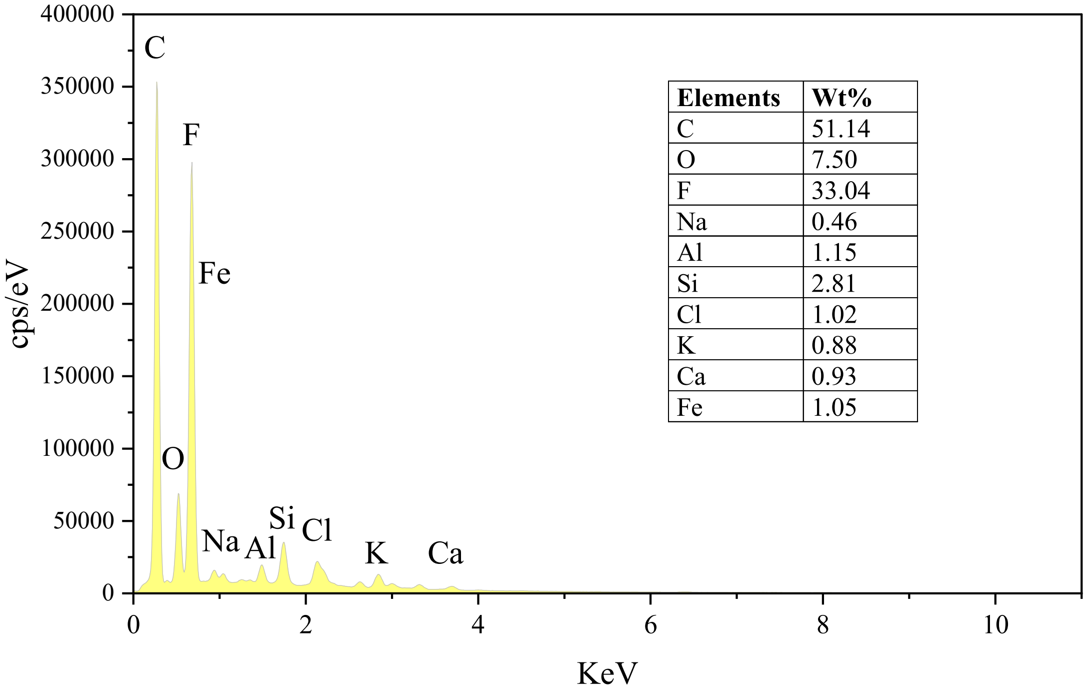

Elements	Wt%
C	51.14
O	7.50
F	33.04
Na	0.46
Al	1.15
Si	2.81
Cl	1.02
K	0.88
Ca	0.93
Fe	1.05

(b)

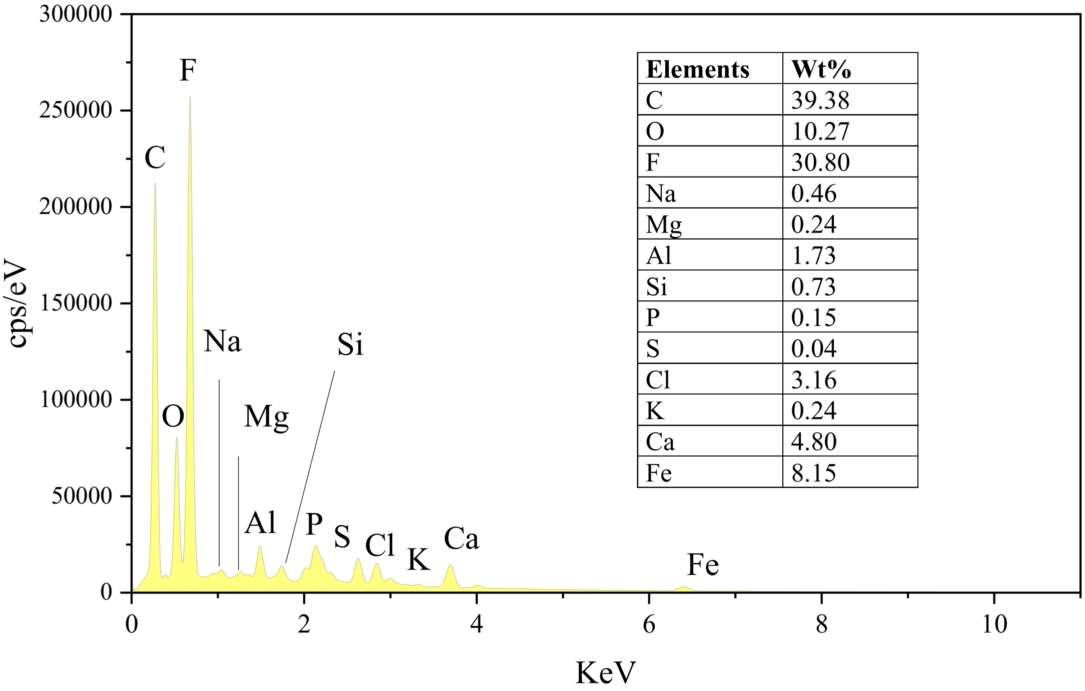

Elements	Wt%
C	39.38
O	10.27
F	30.80
Na	0.46
Mg	0.24
Al	1.73
Si	0.73
P	0.15
S	0.04
Cl	3.16
K	0.24
Ca	4.80
Fe	8.15

(c)

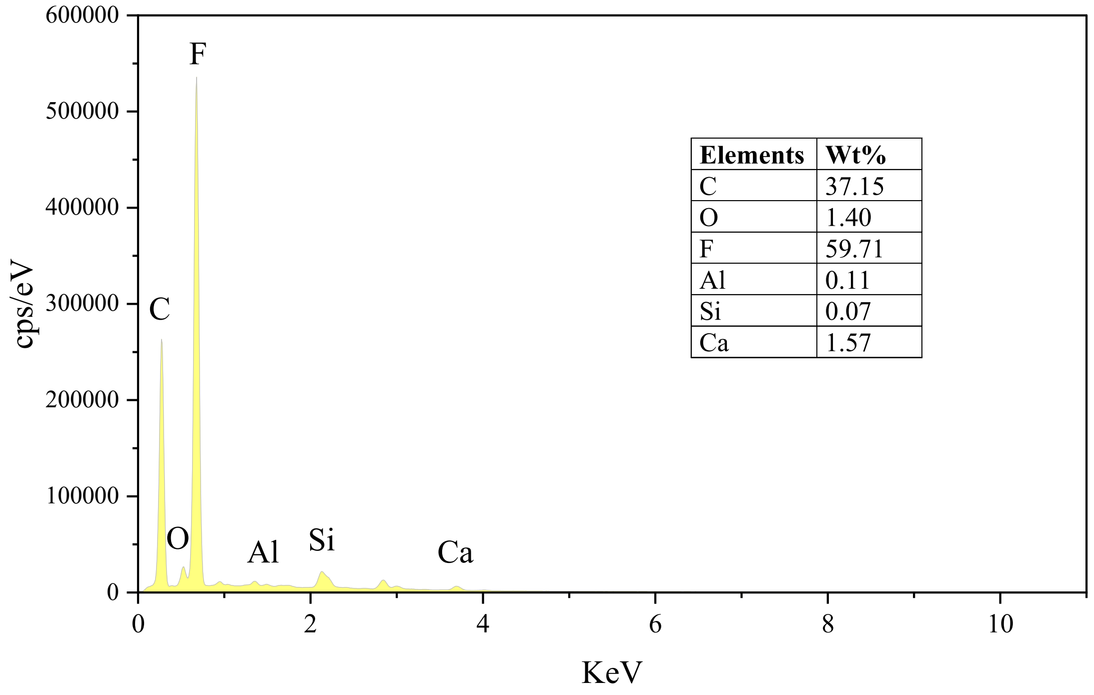

(d)

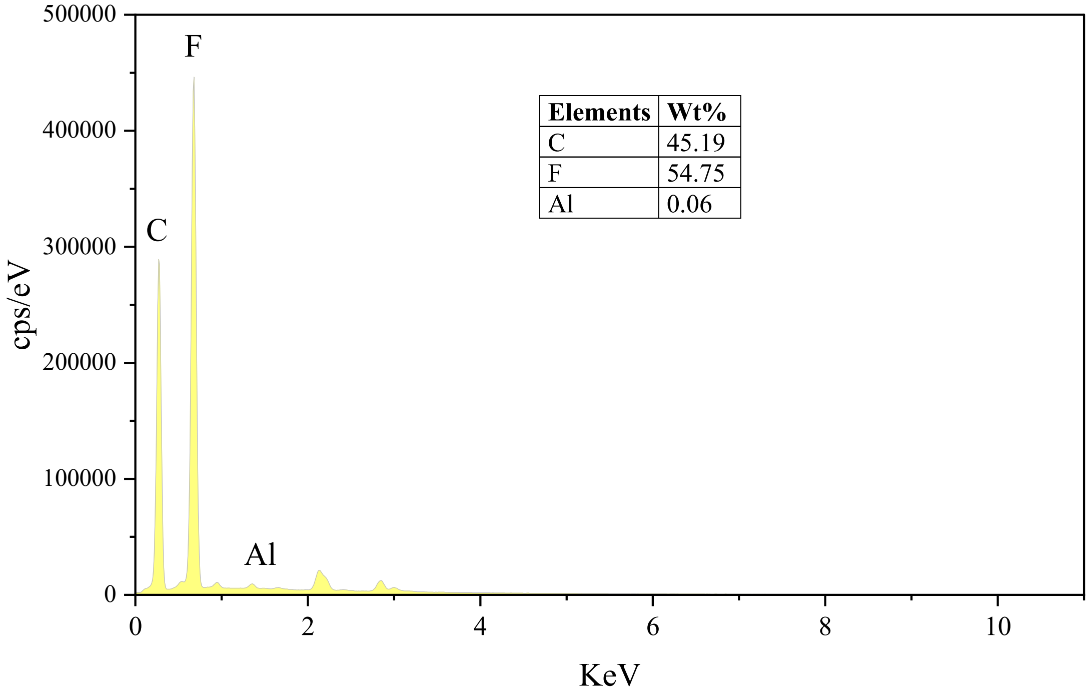

(e)

Figure S4. EDS elemental percentage of (a) virgin membrane, (b) fouled membrane from lab-scale experiment, (c) fouled membrane from pilot run, (d) fouled membrane of leachate treated with tannins, and (e) membrane cleaned with UV+H_2O_2.
